# Ultrafast Cylindrical Vector Beams for Improved Energy Feedthrough and Low Roughness Surface Ablation of Metals

**DOI:** 10.3390/ma16010176

**Published:** 2022-12-25

**Authors:** David Pallarés-Aldeiturriaga, Alain Abou Khalil, Jean-Philippe Colombier, Razvan Stoian, Xxx Sedao

**Affiliations:** 1 Hubert Curien Laboratory, University of Lyon, Jean Monnet University, UMR 5516 CNRS, F-42000 Saint-Etienne, France; 2 GIE Manutech-USD, 42000 Saint-Etienne, France

**Keywords:** femtosecond laser, raster scanning, cylindrical vector beams, raster

## Abstract

The use of ultrafast cylindrical vector vortex beams in laser–matter interactions permits new ablation features to be harnessed from inhomogeneous distributions of polarization and beam geometry. As a consequence, the ablation process can yield higher ablation efficiency compared with conventional Gaussian beams. These beams prevent surface quality degradation during the ablative processes. When processing stainless steel and titanium, the average surface roughness obtained by deploying the cylindrical vector is up to 94% lower than the Gaussian case, and the processing efficiency is 80% higher.

## 1. Introduction

A generation of complex spatial vectorial optical fields has made valuable contributions in various scientific fields, such as encoding information in optical communications, sub-diffraction high-resolution imaging, particle acceleration, and sophisticated control of light–matter interactions [1,2,3,4]. Such states of light can be easily created from a Gaussian beam with nanostructured s-wave-plates [5]. The use of cylindrical vector (CV) beams [6] in ultrafast laser–material ablation can produce complex features, which are both polarization- and fluence-dependent [7,8,9]. Spatially varying polarization states, such as radial and azimuthal polarization, which have a phase singularity and hence an intensity null on-axis, have been used to improve laser cutting, micro-machining [10,11,12,13], and in producing bio-mimetic surfaces [14]. These improvements are related at the same time to the spatial distribution of the intensity and the local dependence of the processing feature on laser polarization. For a given pulse energy, the geometries of CV reduce maximum fluence on the target so that higher pulse energies can be used. In terms of ablation, the CV irradiance distribution can favor plasma ejection as the collision of radially symmetric plasma plume can redirect the momentum of ejected material in the axial direction. Moreover, material processing is strongly dependent of the existing evanescent field, which is created by input laser radiation and scattered surface waves, and their interaction being polarization-dependent [15]. This may have significant consequences in laser processing through raster scanning, where a cumulative and incubative phenomena are at play. The number of laser pulses and evolving state of the surface topography affect the energy coupling of the incoming pulses in a complex way. In this work, ultrafast laser engraving with CV is studied and compared with Gaussian beam engraving. The experiment results suggest that the doughnut shape of the CV beam leads to a better ablation efficiency while the CV polarization distribution appear to prevent surface roughness degradation, and structural light trapping [16].

## 2. Setup and Methodology

**Figure 1:**
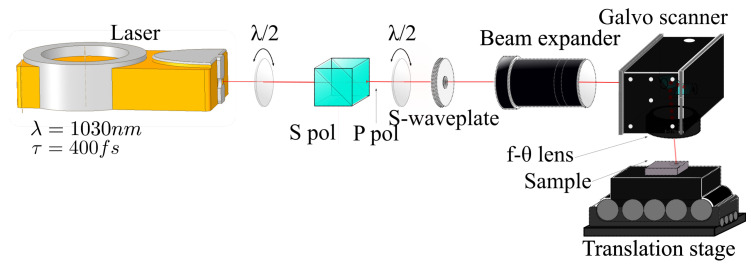
Schematic presentation of the laser inscription setup: s-wave-plate shifts linearly polarized into CV beam and then the scanner focuses the beam onto the sample.

Femtosecond laser engraving is performed using the setup depicted in Figure 1. Here, a femtosecond laser “Tangor HP” from amplitude lasers with a wavelength λ=1030 nm pulse duration τ=400 fs and pulse repetition rate PRR=100 kHz has been employed. The laser beam passes through a half wave plate and a polarizer cube to control laser fluence and then a s-wave-plate (Altechna) to achieve CV beams, then through a beam expander to control the final laser diameter. Then, the shaped beam passes through the galvanometer scanner. A f−θ lens (Ronar-Smith, Winsen, Germany) with f = 170 mm is used to focus the laser beam.

**Figure 2:**
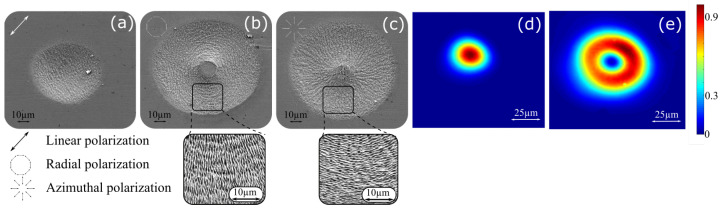
SEM micrographs of impacts on stainless-steel produced by linear Gaussian beam (**a**), azimuthal (**b**), and radial (**c**) polarized doughnut beams at arbitrary pulse energy. They are showing LIPSS perpendicular to polarization direction. Beam profile at focal plane of Gaussian beam (**d**) and doughnut profile with azimuthal and radial polarizations (**e**).

CV beams can be obtained using a s-wave-plate. The laser beam out from the laser cavity is linear polarized light whose polarization direction can be controlled through a half wave-plate. When both the polarization and the s-wave-plate axis are parallel, radial polarization is achieved while a perpendicular orientation generates azimuthal polarization. These CV states have been double-checked by launching pulses to a stainless-steel surface and studying Laser Induced Periodic Surface Structures (LIPSS) orientation. Figure 2a shows an impact produced by a circular Gaussian beam with a linear polarization. LIPSS are perpendicular to the polarization direction as expected [14]. In radial/azimuthal distribution, LIPSS appear to be concentric and radially aligned, respectively, as depicted in Figure 2b,c.

When a Gaussian beam is converted to CV, the Gaussian intensity distribution shifts also to a doughnut shape, which in terms of fluence, a fits the following function [17]:

(1) $$F\left(r\right)=\frac{2}{\omega_{0}^{2}}F_{0}r^{2}e^{\left(1-\frac{2r^{2}}{\omega_{0}^{2}}\right)}$$

where $F_{0}$ is the peak fluence at $r=\omega_{0}/\sqrt{2}$ and $\omega_{0}$ is a fitting parameter. This original Gaussian beam and doughnut profiles were measured by a Gentec-EO camera Beamage-4M with a magnifying system. Both are depicted in Figure 2d,e exhibiting a beamwaist and fitting parameter of 16.4 and 20.3 µm, respectively. The effective beam area (EBA) of the doughnut using the 1/e^2^ criterion is 3.9 times higher than the Gaussian one, which makes $F_{0}^{rad/azi}=0.26F_{0}^{linear}$. This change in beam diameter also changes $N_{eff}$ when the same scanning speed is employed during the inscription. $N_{eff}$ can be defined as the average value of the sum of normalized fluence distributions in a laser raster scanning inscription [17], which for the 2D Gaussian case is:

(2) $$\begin{array}{cc} {N_{eff}^{max}}_{G} & ={\left(\sum_{k}\exp\left(-8{\left(\left(1-\phi \right)k\right)}^{2}\right)\right)}^{2} \end{array}$$

(3) $$\begin{array}{cc} {N_{eff}^{min}}_{G} & ={\left(\sum_{k}\exp\left(-8{\left(\left(1-\phi \right)\left(1/2+k\right)\right)}^{2}\right)\right)}^{2}. \end{array}$$

(4) $$\begin{array}{cc} {N_{eff}}_{G} & =({N_{eff}^{max}}_{G}+{N_{eff}^{min}}_{G})/2 \end{array}$$

where ϕ is the overlap ratio and can be defined as a function of the consecutive pulse distance Λ and $\omega_{0}$: $\left(1-\phi \right)=\Lambda /2\omega_{0}$. Here $\Lambda =\Lambda_{x}=\Lambda_{y}$ and the number of pulses per line *k* is equal to the number of lines. In the case of radial/azimuthal polarization, Equations (2) and (3) are rewritten using Equation (1):

$$\begin{array}{cc} {N_{eff}^{max}}_{CV}= & 8e\sum_{k_{x}}\sum_{k_{y}}{\left(1-\phi \right)}^{2}\left({\left(1/2+k_{x}\right)}^{2}+{\left(1/2+k_{y}\right)}^{2}\right) \end{array}$$

(5) $$\begin{array}{cc} \; & \exp(-8{\left(1-\phi \right)}^{2}\left({\left(1/2+k_{x}\right)}^{2}+{\left(1/2+k_{y}\right)}^{2}\right)) \end{array}$$

(6) $$\begin{array}{cc} {N_{eff}^{min}}_{CV}= & 8e\sum_{k}\sum_{k}{\left(1-\phi \right)}^{2}\left(k_{x}^{2}+k_{y}^{2}\right)\exp\left(-8{\left(1-\phi \right)}^{2}\left(k_{x}^{2}+k_{y}^{2}\right)\right) \end{array}$$

where *k_x_* is the number of pulses per line and *k_y_* is the number of lines. An overlap ratio of ϕ=0.7 has been chosen as based on previous studies of process optimization using Gaussian laser beams [18]. Applying Equation (4), ${N_{eff}}_{G}=4.36$. Keeping same scan speed, the larger beam area of CV beams shifts the overlap ratio to ϕ=0.76 and using Equations (5) and (6) the new effective pulse number is ${N_{eff}}_{CV}=18.53$. The accumulated fluence (or accumulated dose) can be defined as $\Gamma =N_{eff}F_{0}$. Therefore, when scanning speed is kept constant, the pulse energy must be adapted in order to achieve identical accumulated fluence by a ratio ${E_{0}}_{G}=1.09{E_{0}}_{CV}$. This value is very close to unity, which implies that it is similar to a same average power situation for both beam types.

Engraving performance has been studied by laser raster scan inscribing 2 × 4 mm rectangle areas. In order to achieve deep patterns (depth > 100 µm), the number of scans required (for linear polarization) was previously determined in a preliminary test. Distance between pulses $\Lambda_{x}$ for one scan and line distance $\Lambda_{y}$ were equalized. Once inscriptions have been performed, the depth and roughness were measured with a chromatic confocal microscope (Altimet, Stil). For qualitative observation of surface texture at the microscale, a scanning electron microscope (SEM, Jeol) was used. Finally, as an additional ablation efficiency indicator, the differential weighting method has also been performed [19]. Stainless steel 316 L and titanium were purchased from Neyco Vacuum and Materials, the initial roughness was Ra≈0.4 µm for stainless steel and Ra≈1.6 µm for titanium.

In order to compare Gaussian beam ablation with doughnut-shaped CV beams ablation, static laser irradiation, 1D scanning irradiation, and 2D raster scanning were carried out.

## 3. Results

In static mode, static shots at the same fluence (hence identical accumulated fluence) were delivered to the sample for a range from 2 to 10 J/cm^2^. In terms of ablation depth, there is no marked difference between Gaussian and CV beams (it is noted that the crater depth for CV beam ablation is achieved in the annular ring region but not in the center [20]).

**Figure 3:**
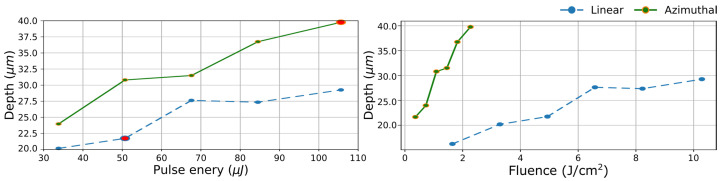
Depth variation as a function of the pulse energy (**right**) and fluence (**left**) of inscribed lines for Gaussian linear polarization and CV azimuthal polarization at 1 mm/s. Lines inscribed with linear polarization exhibit consistently lower depth than the ones inscribed with azimuthal polarization. The bold data points representing identical accumulated fluence.

Furthermore, in order to compare the effect of Gaussian and azimuthal polarization in 1D laser engraving, lines (120 scans) were inscribed with pulse energies ranging from 34 to 105 µJ. The result is depicted in Figure 3 (left) at a fixed laser pulse energy, lines inscribed by CV with azimuthal polarization are consistently deeper than the ones inscribed by Gaussian. Two conditions with identical accumulated fluence are also highlighted in Figure 3 (left) (the bold data points for linear Gaussian and for CV). When accumulated fluence is concerned, the difference in depth is even more significant, almost a factor of 2. This trend is also conserved when values are rearranged in function of regular fluence as depicted in Figure 3 (right).

**Figure 4:**
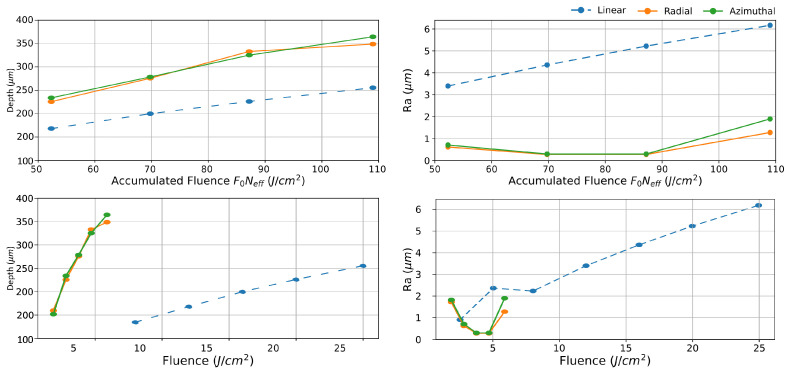
Depth (**right**) and arithmetic roughness (**left**) against accumulated fluence (**top**) and regular fluence (**bottom**) for linear Gaussian and CV in stainless-steel. CV produces significant lower roughness compared to the linear Gaussian case, being 16 times smaller at intermediate fluences. Besides, they produce deeper pattern by a factor two compared by Gaussian distribution.

Next, 2D pattern engraving is investigated. For the same accumulated fluence study in a raster scanning situation, the methodology described in Section 2 is used. In this way, rectangles were inscribed from 8 to 25 J/cm^2^ for Gaussian shape, and from 1.9 to 5.9 J/cm^2^ for the CV in doughnut shape. Scan speed was set to 1 m/s, which corresponds to 70% overlap for Gaussian. This allows an identical accumulated fluence Γ varying from 52 to 109 J/cm^2^ per scan (150 scans in total) in both cases. Figure 4 (left) depicts the depth variation of both CV compared with Gaussian distribution. Here, the ablation with CV distributions produces greater engraving depth, and consequently higher efficiency compared with Gaussian. This engraving depth enhancement by a 20% is observed at low fluence end, and it increases up to 42% at Γ=109 J/cm^2^. In this way, inscription with CV outperforms Gaussian linear polarization with energy increment.

**Figure 5:**
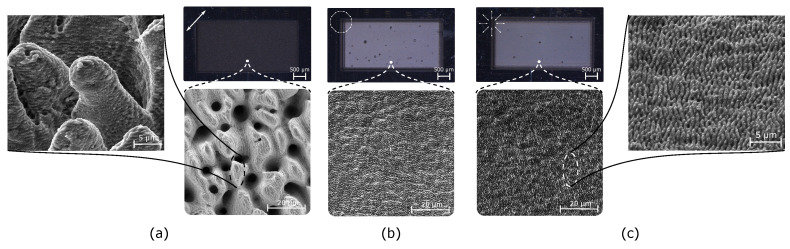
Optic microscope (top) and zoomed SEM micrographs (bottom) of engraved stainless-steel rectangles at 1 m/s scan speed at 51.3 µJ and 100 kHz for: linear Gaussian (**a**), and CV with azimuthal (**b**) and radial (**c**) polarizations.

Concerning the surface roughness, Figure 4 (right) depicts roughness evolution with accumulated fluence. When CV beams are employed, the surface roughness is drastically reduced. However, while its behavior with Gaussian beams is linear with fluence, with CV beams, roughness exhibit an irregular behavior with a local minima at intermediate fluences (70–87 J/cm^2^). Here, roughness can be as low as 0.28 µm which is 19 times lower than the Gaussian counterpart. Nonetheless, at lower fluences, the average roughness is slightly compromised, presumably due to the increased number of surface irregularities in the laser-irradiated area, such as the ones shown in Figure 5. It is hypothesized that, at the low-fluence regime, 50–70 J/cm^2^, as is the case here, surface impurities and/or random surface reflections may play an important role, causing surface irregularities to form during the ablation process [21,22]. The formation mechanism of this micro-cavities is under investigation and will be reported in a separate study. On the other hand, at high fluence end, the surface roughness increases slightly with fluence, but is still far better than the surface roughness achieved in the Gaussian case. Figure 4 (below) depicts both depth and roughness evolution with regular fluence where the high performance of CV is maintained even for roughness where pulse energy of CV beams is four times higher than for Gaussian beams. This out-performance of CV over linear Gaussian beam in engraving could be explained tentatively as follows: the early laser scans for engraving produces an initial corrugation that induces a polarization dependent scattering. In the Gaussian case, the evolving corrugation by successive laser irradiation scans is further amplified during laser scan due to amplified scattering by the anisotropic linear laser polarization. On the other hand, for CV, a non-uniform polarization smears directional scattering, and provides lower roughness.

These results suggest that CV might be employed to achieve high quality engraving at high laser power (thus, less processing time). A showcase is made on engraving of stainless steel and titanium, which were shown to be difficult to deal with at high laser power conditions [23]. Here, both materials were inscribed with the same parameters. The samples were inscribed at 1 m/s. The pulse energy was set to 51.3 µJ in all cases which represents fluences of 12 and 3 J/cm^2^ for Gaussian and for CV configurations, respectively. The number of scans for titanium was 180 and 150 for stainless steel. Figure 5 illustrates the engraving results for stainless steel. It comes clear that (for same power) CV engraving with radial and azimuthal polarization states produces a more regular, smoother and hence shinier surface. These observations agree with the confocal evaluation, where an average surface roughness of 3.87 µm for Gaussian engraving has been measured. The surface roughness of CV with radial and azimuthal ones is 0.22 and 0.21 µm, respectively, which features a 94% roughness reduction, compared to Gaussian engraving case. The 3 samples were additionally examined using SEM to study the types of texturing patterns. The sample irradiated with Gaussian linear polarization exhibits a bumpy surface morphology that favors light trapping [16]. However, in the case of CV with radial polarization, the surface becomes more leveled, with homogeneous LIPSS of dramatically lower depth variation. Azimuthal polarization produces similar surface morphology where LIPSS are perpendicularly oriented to the radial case.

Titanium samples engraving shows similar behavior to stainless-steel engraving. The surface engraved by Gaussian is characterized by a bumpy appearance with high surface roughness, just like the case of stainless-steel engraving with Gaussian. The engraving characterization with depth, mass removal, and surface roughness for the two materials in question is summarized in Table 1. One can see that by employing CV with radial/azimuthal polarization, the inscription depth increases by 130%, the mass removal increases by 100%, and surface roughness is reduced by 96% compared with the inscription with linear Gaussian beam. These improvements are slightly better for titanium than for stainless steel for the same parameters. These two examples imply that in an industrial setting fast and high quality engraving can be achieved by using CV beam, without the need to attenuate laser output power. In a separate study, to benchmark the engraving quality, a bigger Gaussian beam with spot size similar to the CV beam was applied at the same process condition; neither process yield nor surface roughness was comparable to CV beam engraving. This unique performance enhancement by CV might be attributed to two possible factors. First, it could be caused by a higher ablation efficiency caused by a more efficient evacuation of plasma plume from the central hollow of the doughnut geometry [20]. Second, it could be caused by the non-homogeneous polarization distribution, which leads to a more homogenized surface wave scattering, which constrains surface roughness/corrugation amplification during the laser process [24], preventing roughness increment. These effect cannot be mentioned yet as dominant in the present experiments. For a better understanding, further experiment is envisaged to de-correlate the role of doughnut geometry and CV polarization.

## 4. Conclusions

The advantages of laser processing, originating in the use of CV beams, have been showcased; the enhanced process yield and surface roughness after ablation have been demonstrated. Both doughnut beam geometry and CV polarization (radial/azimuthal) seem to play a role in the complex interplay of laser–material interactions. The doughnut beam geometry is likely to contribute to a more efficient energy coupling, while its polarization uniformity leads to less light confinement and, hence, a smoother surface profile.

## Figures and Tables

**Table 1:** Ablation efficiency (volume ablated per pulse); mass removal and Ra for titanium and stainless steel at same power.

black	Parameters	Pol	δ v/pulse (μm^3^)	δ m (mg)	Ra (μm)
Titanium	51.4µJ	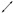	58.5	6.08	3.65
	1 m/s	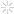	135.7	11.76	0.135
	×180	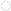	138.9	12	0.15
Steel	51.4 µJ	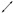	60.2	9.18	3.87
	1 m/s	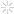	106.6	15.59	0.22
	×150	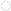	108.3	15.95	0.21

## Data Availability

Not applicable.
